# Effect of a single dose of pregabalin on herpes zoster pain

**DOI:** 10.1186/1745-6215-12-55

**Published:** 2011-02-28

**Authors:** Christina Jensen-Dahm, Michael C Rowbotham, Haatem Reda, Karin Lottrup Petersen

**Affiliations:** 1 Department of Neurology University of Copenhagen, Rigshospitalet, Denmark; 2 California Pacific Medical Center Research Institute, CA, USA; 3 Department of Neurology, Mayo Clinic, Rochester, Minnesota, USA; 4 University of California San Francisco, Department of Neurology, CA, USA

## Abstract

**Background:**

The effect of pregabalin on acute herpes zoster pain has not been previously evaluated.

**Methods:**

In a randomized, double-blind, placebo-controlled, two-session crossover study the effect of a single oral dose of pregabalin (150 mg) on pain and allodynia was evaluated in 8 subjects with herpes zoster.

**Results:**

Over 6 hours of observation, pain decreased by a mean of 33% with pregabalin and 14% with placebo (p < 0.10). Effects on allodynia and SF-MPQ were not significant.

**Conclusions:**

Compared to an earlier study of gabapentin 900 mg for acute zoster pain and allodynia that followed a nearly identical protocol, pregabalin had a similar effect on pain and was well tolerated, with no difference from placebo on sleepiness. Common side effects of light-headedness, unsteady gait, and slowed thinking were almost identical to that observed in the earlier study of gabapentin. Subject recruitment proved difficult in part due to the widespread off-label use of gabapentin and pregabalin for acute zoster pain in our region of the USA.

**Trial Registration:**

ClinicalTrials.gov Identifier: NCT00352651

## Introduction

Herpes zoster (HZ), the reactivation of latent varicella zoster virus in sensory ganglia, produces a painful, unilateral, dermatomal rash. We have previously demonstrated that a single oral dose of gabapentin (900 mg) reduces pain and allodynia during HZ [1]. Both pregabalin and gabapentin bind to the α2δ-subunit of the calcium channel [2], but no head-to-head efficacy comparisons have been made. In this randomized, placebo-controlled, two-session, crossover study we evaluated the effect of a single dose of 150 mg of pregablin on pain and allodynia in subjects with HZ using a nearly identical protocol.

## Materials and methods

### Subjects

All subjects provided informed written consent. The study was approved by the UCSF Committee on Human Research. Subjects were medically stable adults with onset of unilateral HZ during the previous 6 weeks and continuing pain ≥ 40 on a 100 mm pain visual analogue scale (VAS; 0 = no pain, 100 = worst imaginable pain) at the screening visit. Exclusion criteria included: prior pregabalin use, nerve block therapy within the last 48 hours, use of gabapentin within the last 72 hours, unrelated severe pain and clinically significant medical or psychiatric disease. Stable doses of oral NSAIDs, acetaminophen, opioids, anticonvulsants (except pregabalin or gabapentin) or antidepressant were allowed and could be taken up to 2 hours prior to medication visits. Subjects were not allowed to use topical analgesics 12 hours prior to medication visits.

### Visits and measures

Following the screening visit, the two medication visits (V1 and V2) were separated by at least 48 hours. Medication was administered double-blind as a single dose of 150 mg of pregabalin or identical-appearing placebo. Randomization was computer generated and managed by a study pharmacist not otherwise involved in the study. Subjects rated their pain on the 100 mm pain VAS. Allodynia area was mapped by stimulating the skin with a 1-inch foam brush going from normal to affected skin. Within the area of greatest pain, the intensity of allodynia on a 100 mm allodynia VAS scale (0 = not unpleasant, 100 = most unpleasant imaginable) was rated after 3 strokes with the foam brush. Subjects rated side-effects (sleepiness, light-headedness or dizziness, unsteady gait, nausea and vomiting) on a 0-3 scale (0 = none, 1 = mild, 2 = moderate, 3 = severe). Pain, allodynia intensity and side-effects were rated prior to medication and every 30 minutes for 6 hours. Allodynia areas were mapped at 1,2,3, and 6 hours post-medication. The Short Form McGill Pain Questionnaire was administered pre-medication and 2 hours post-medication [3].

### Statistical analysis

In a previous study a single 900 mg dose of oral gabapentin reduced HZ pain by approximately 33% (compared to placebo). The standard deviation of the change was 50%, resulting in a standardized effect size (E/S) of 0.7. With a two-tailed α of 0.05, and a β of 0.2, the sample size desired is 34. The primary efficacy variable, VAS pain, was analyzed using a model that included fixed effects for treatment, age, treatment order, sex, and baseline pain and a random subject effect to account for correlated data between the two treatments. A similar analysis was used for other, secondary, repeated measures. Post hoc analyses of percent reduction from baseline at each time point for the primary efficacy variable used the Wilcoxon signed-rank test. SF-MPQ (total score) results were analyzed with Wilcoxon signed-rank test.

## Results

**Figure 1:**
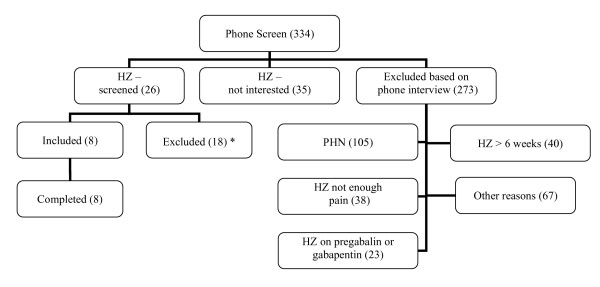
**CONSORT Statement**. * Screened subjects excluded before randomization due to: not enough pain (1), BMI < 20 (1), creatinine clearance <60 ml/min (2), significant medical or psychiatric illness (4), medication (1), unclear diagnosis (1), withdrew consent (8).

**Figure 2:**
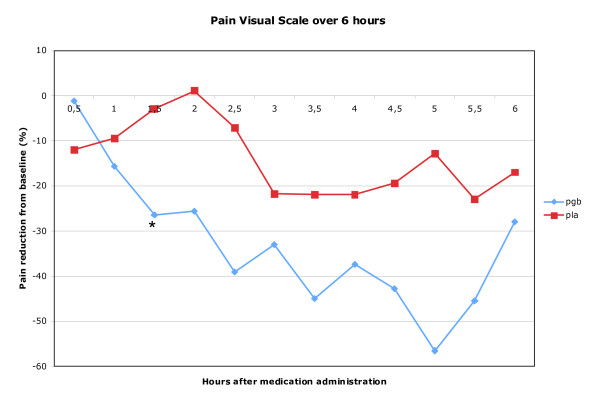
**Percent reduction in median pain VAS (placebo and pregabalin) at specific time points after medication administration**. A larger negative number means greater reduction in pain. * p < 0.05.

Between January 2006-March 2008, 334 potential subjects were telephone screened, with 26 attending a screening visit (Figure 1). Eight subjects were dosed and all completed the study. The study was closed in March 2008 due to the slow pace of enrollment. Baseline characteristics are shown in Table 1 and changes in pain ratings during the two drug administration sessions are shown in Figure 2. Overall, there was a trend toward greater reduction in pain after pregabalin administration than after placebo (placebo - pregabalin: -19.0% [95% CI: -42.5% to +4.5%]; p = 0.096. Analysis of the individual time points for the difference between pregabalin and placebo revealed significance at 1.5 hours after medication administration (-23.5% [- 44.5% to -2.5%]; p = 0.034), and trends at 2.5 hours (-31.9% [-70.1% to +6.3%]; p = 0.099) and 5 hours (-43.7% [-91.2% to +3.9%]; p = 0.076). Relative to placebo, we found a trend towards greater maximal pain reduction with pregabalin (-22.9% [-52.0 to + 6.0); p = 0.100). Changes in severity and area of allodynia, and the change in SF-MPQ scores, were not significantly different between the pregabalin and placebo sessions (data in Table 2). Side-effects were well tolerated (Table 2).

**Table 1:** Baseline demographics, pain and allodynia.

	Placebo first group, n = 3	Pregabalin first group, n = 5
Demographics		

Age; median (range)	77 (73-84)	65 (22-76)

% Female	33%	40%

Distribution of Zoster		

Trigeminal	0	1

Cervical	1	1

Thoracic	2	3

Severity of rash		

Mild	0	0

Moderate	100% ^a^	80%

Severe		20%

Days between outbreak and study entry; median (range)	31 (31-37)	23 (10-45^b^)

Days between visit 1 and visit 2; median (range)	2 (2-5)	2 (2-5)

Pain at V1, 0-100; median (range)	63 (20-90)	48 (16-90)

Pain at V2, 0-100; median (range)	55 (17-79)	30 (9-94)

Allodynia severity V1, 0-100; median (range)	51 (19-74)	37 (10-84)

Allodynia severity V2, 0-100; median (range)	20 (15-48)	34 (0-70)

^a ^One subject missing, ^b ^One subject included at 45 days; met all other inclusion criteria.

**Table 2:** Outcome Measures

Outcome (Mean and 95% CI)	Pregabalin, n = 8	Placebo, n = 8
Overall Pain percent reduction	33.0 (13.2 - 52.9)	14.0 (-5.8 to 33.8)

Overall Pain VAS reduction	14.7 (5.1 - 24.3)	8.5 (-1.1 to 18.1)

Allodynia severity, overall percent reduction	25.2 (0.9 - 49.5)	30.7 (7.5 - 53.9)

Allodynia area, overall percentage reduction	-1.4 (-59.6 to 56.8)	-12.4 (-70.8 to 45.9)

Total SF McGill (0-45), premedication	12.4 (8-25)	12.3 (7-26)

Total SF McGill (0-45), post medication	7.25 (3-22)	8.9 (6-16)

Side-effects; 0 to 3		

Sleepiness	0.7 (0-1.2)	0.7 (0-1.7)

Light-headedness	0.5 (0-1.6)	0.2 (0-1.1)

Unsteady gait	0.2 (0 -1.2)	0.05 (0-0.4)

Slowed thinking	0.2 (0-1)	0

Headache	0.01 (0-0.08)	0.1 (0-0.5)

Nausea	0.1 (0-1)	0.09 (0-0.4)

Vomiting	0	0

Blurry vision	0.01 (0 -0.08)	0

Total Side-effect score	1.7 (0.2 - 3.9)	1.1 (0-4.1)

## Discussion

Pregabalin has not been previously studied for acute zoster pain. Pregabalin has been studied in Post Herpetic Neuralgia (PHN), the chronic neuropathic pain condition afflicting some patients after an episode of herpes zoster (HZ). In multiple clinical trials for PHN, pregabalin has been proven effective at daily doses of 150 mg/day and higher [4,5]. Efficacy is demonstrable within a few days of starting treatment [4]. In one large trial, changes in allodynia severity correlated with changes in ongoing pain [5]. Despite the very small number of subjects in the present study, there was a trend towards a greater reduction in pain after a single dose of 150 mg of pregabalin. Using the data given in table 2, the standardized effect size is 1.16. With this effect size and a two-sided α of 0.05 and β of 0.2, a future study would need only 18 subjects to reach significance. A significant effect of pregabalin on allodynia severity or area was not demonstrated.

A previous study of nearly identical design conducted in our unit included 26 subjects with HZ and showed that a single dose of 900 mg gabapentin reduced both pain and allodynia [1]. In the present study, the maximum percent reduction in pain with pregabalin was 63% compared to 40% with placebo, quite similar to the 66% reduction with gabapentin and 33% reduction with placebo in the earlier study. The pattern of side effects was quite similar between the two studies, with the noteworthy exception that sleepiness was greater than placebo with gabapentin, and identical to placebo with pregabalin. Trials using a single relatively large dose have only limited ability to predict longer term treatment results. The recent multicenter trial by Dworkin and colleagues in 87 subjects treated within 6 days of zoster rash onset compared placebo, oxycodone, and gabapentin up to 600 mg three times a day [6]. Gabapentin therapy showed a trend for efficacy in the first week which was completely lost as the trial continued, while pain reduction with oxycodone remained significantly greater than with placebo. However, the natural history of the decline in pain complicates longer-term trials in HZ patients. At treatment day 9 in the Dworkin et al study, even 'worst pain' in the placebo group was already below 4/10.

When evaluating efficacy of new compounds for neuropathic pain in HZ and PHN, baseline pain ratings should be at least moderate to severe to allow measurement of pain relief. Herpes zoster is much more common than PHN, with well over a million new cases each year in the USA, and in the study of Helgason and colleagues, between 7%-30% of subjects reported their acute zoster pain as moderate to severe [7]. The incidence of moderate to severe PHN after HZ is lower; in the landmark vaccine study by Oxman and colleagues, the incidence of PHN (worst pain >/= 3/10) was 5.1% at 6 months in placebo vaccinated subjects aged 60 or older [8]. However, as PHN pain is chronic and often insufficiently treated, the point prevalence of patients looking to participate in clinical trials may indeed be larger than the point prevalence of patients with moderate-severe HZ pain, many of whom are hoping the pain will spontaneously subside. When considering whether to study HZ or PHN, it should also be kept in mind that the neurodestructive and inflammatory aspects of herpes zoster might make this condition respond differently to treatment than chronic neuropathic pain disorders such as PHN and painful diabetic neuropathy.

Recruitment is always an important issue in clinical trials, and this study demonstrates that recruitment challenges can be severe. During an enrolment period of more than 2 years, we phone screened 334 subjects yet dosed a total of 8. Only 162 were within the specified time frame after rash onset for study inclusion and others had insufficient pain. Unlike trials of investigational compounds not otherwise available, many otherwise eligible subjects declined to participate because pregabalin could be obtained from their primary physician, or they had current or prior treatment with pregabalin or gabapentin for their HZ pain (frequently initiated at the time of zoster diagnosis). We used only print newspaper and online advertising (Craigslist). Recruiting within emergency rooms, primary care clinics, and dermatology practices might yield untreated and potentially eligible subjects within the first few days of rash onset.

## Conclusions

In summary, this small placebo-controlled crossover trial of a single relatively large dose of pregabalin showed a trend for reducing acute zoster pain. The effect size was similar to that observed in a prior study of gabapentin in a much larger group of acute zoster patients. Greatly complicating recruitment for this placebo-controlled study was the widespread clinical practice of 'off-label' initiation of pregabalin or gabapentin therapy at the time of zoster diagnosis.

## Competing interests

The authors declare that they have no competing interests.

## Authors' contributions

CJD conducted the study and drafted the manuscript. KLP designed the study, performed the statistical analysis and helped draft the manuscript. MCR was study physician, participated in study design, and helped draft the manuscript. HR helped design and conduct the study. All authors read and approved the final manuscript.
